# Genome wide analysis of gene expression changes in skin from patients with type 2 diabetes

**DOI:** 10.1371/journal.pone.0225267

**Published:** 2020-02-21

**Authors:** Eri Takematsu, Adrianne Spencer, Jeff Auster, Po-Chih Chen, Annette Graham, Patricia Martin, Aaron B. Baker

**Affiliations:** 1 University of Texas at Austin, Department of Biomedical Engineering, Austin, TX; 2 Department of Biological and Biomedical Sciences, School of Health and Life Sciences, Glasgow Caledonian University, Scotland, United Kingdom; 3 Institute for Cellular and Molecular Biology, University of Texas at Austin, Austin, TX; 4 The Institute for Computational Engineering and Sciences, University of Texas at Austin, Austin, TX; 5 Institute for Biomaterials, Drug Delivery and Regenerative Medicine, University of Texas at Austin, Austin, TX; University of Edinburgh, UNITED KINGDOM

## Abstract

Non-healing chronic ulcers are a serious complication of diabetes and are a major healthcare problem. While a host of treatments have been explored to heal or prevent these ulcers from forming, these treatments have not been found to be consistently effective in clinical trials. An understanding of the changes in gene expression in the skin of diabetic patients may provide insight into the processes and mechanisms that precede the formation of non-healing ulcers. In this study, we investigated genome wide changes in gene expression in skin between patients with type 2 diabetes and non-diabetic patients using next generation sequencing. We compared the gene expression in skin samples taken from 27 patients (13 with type 2 diabetes and 14 non-diabetic). This information may be useful in identifying the causal factors and potential therapeutic targets for the prevention and treatment of diabetic related diseases.

## Introduction

Type 2 diabetes affects 29 million people in the U.S., and 170 million people in the world[1]. This condition can often lead to the disturbance of the blood vessel wall through promotion of vascular inflammation and endothelial cell dysfunction[2, 3]. These abnormalities increase the severity of vascular disease in diabetic patients[4]. A major complication of diabetes is the formation of non-healing ulcers. Patients with type 2 diabetes are prone to the development of non-healing ulcers, particularly on the lower limbs[5]. These non-healing ulcers are a major factor in the cost of treating diabetes. One estimate suggested that non-healing ulcers add 9–13 billion dollars to the overall annual cost of treating diabetes in the United States alone[6]. A wide variety of treatments have been explored to heal or prevent these ulcers from forming[7]. However, the majority of these treatments have been found to either be ineffective in clinical trials or have limited benefits in a subset of patients[8].

Our group has recently identified that patients with diabetes have a reduction in cell surface proteoglycans in their skin, including syndecan-4 and glypican-1[7, 9, 10]. These proteoglycans serve as co-receptors for growth factor signaling and their absence would suggest that diabetic tissues would be resistant to growth factor therapies for enhancing angiogenesis and wound healing. Delivery of syndecan-4 and glypican-1 in a proteoliposomal formulation enhanced the effectiveness of growth factor therapies in diabetic animals in models of limb ischemia and wound healing[7, 10]. These studies demonstrated that an increased understanding into the biology of non-healing ulcers and the changes that occur in skin with type 2 diabetes could identify potentially treatable deficits in healing and angiogenesis that may improve their treatment.

In this work, we used RNA sequencing (RNAseq) to examine the changes in gene expression in the skin between patients with type 2 diabetes and non-diabetic patients. These findings provide a window into the changes that occur in diabetic patients that may predispose them to the formation of non-healing ulcers and could provide pathways for further investigation into the mechanisms of poor healing in diabetic wounds.

## Methods and materials

### Human samples

Human skin samples were obtained from the Glasgow Caledonian University Skin Research Tissue Bank, Glasgow UK. The tissue bank has NHS research ethics approval to supply human skin for research (REC REF: 16/ES/0069). All methods were carried out in accordance with relevant guidelines and regulations. All experimental protocols were approved by the NHS East of Scotland Research Ethics Service. Informed consent was obtained from all subjects (no patients were under 18 years of age). All the patients were Caucasian and from western Scotland. Patient with diabetes were on treatment with metformin. Samples were formalin fixed and embedded in paraffin following standard procedures prior to sectioning. Control skin samples were taken from either clinical cases of breast reduction surgery or lower limb interventions for peripheral arterial disease. Skin sample for diabetic patients were taken from during limb amputation surgeries. Metadata for the patients are listed in **Table 1**.

**Table 1:** Patient metadata.

Sex	Age	Diabetic State	Sample Location
M	59	Normal	Lower Limb
F	49	Normal	Lower Limb
F	49	Normal	Lower Limb
M	69	Normal	Lower Limb
M	69	Normal	Lower Limb
F	80	Normal	Lower Limb
F	39	Normal	Breast
M	62	Normal	Lower Limb
F	19	Normal	Breast
F	34	Normal	Breast
F	60	Normal	Breast
F	27	Normal	Breast
F	69	Normal	Breast
M	73	Type II Diabetic	Lower Limb
F	47	Type II Diabetic	Lower Limb
M	80	Type II Diabetic	Lower Limb
M	81	Type II Diabetic	Lower Limb
M	53	Type II Diabetic	Lower Limb
M	77	Type II Diabetic	Lower Limb
M	61	Type II Diabetic	Lower Limb
M	79	Type II Diabetic	Lower Limb
M	61	Type II Diabetic	Lower Limb
M	49	Type II Diabetic	Lower Limb
M	58	Type II Diabetic	Lower Limb
F	72	Type II Diabetic	Lower Limb
F	59	Type II Diabetic	Lower Limb
F	59	Type II Diabetic	Lower Limb

### RNA sequencing and analysis

RNA was isolated from formalin-fixed, paraffin-embedded tissue sections using the RNeasy FFPE kit (Qiagen). The mRNA was sequenced using an Illumina HiSeq 4000 at the Genomic Sequencing and Analysis Facility at UT Austin. Single reads of 50 base pairs were performed after poly-A mRNA capture used Ambion Poly(A) Tailing Kit and NEBNext Ultra II Directional RNA Library Prep Kit to isolate mRNA and dUTP directional preparation of the mRNA library. Gene expression analysis was performed using DESeq2 and R software. Plots were created using Prism GraphPad and Microsoft Excel.

### Statistical analysis

Gene abundances were then normalized using DESeq2 normalization and log2 transformed using variance-stabilizing transformation (VST). Differential expression testing was performed based on the DESeq2 negative binomial distribution by comparing type II diabetic samples to normal samples, while controlling for sex differences. This was done by including sex as a covariate to control for in the DESeq2 design matrix along with the factor of interest, diabetic state. Genes that met the adjusted p-value cutoffs of 0.05 or lower and an absolute fold change of 2 or higher.

## Results and discussion

### Overall changes in gene expression in the skin in type 2 diabetes

**Fig 1:**
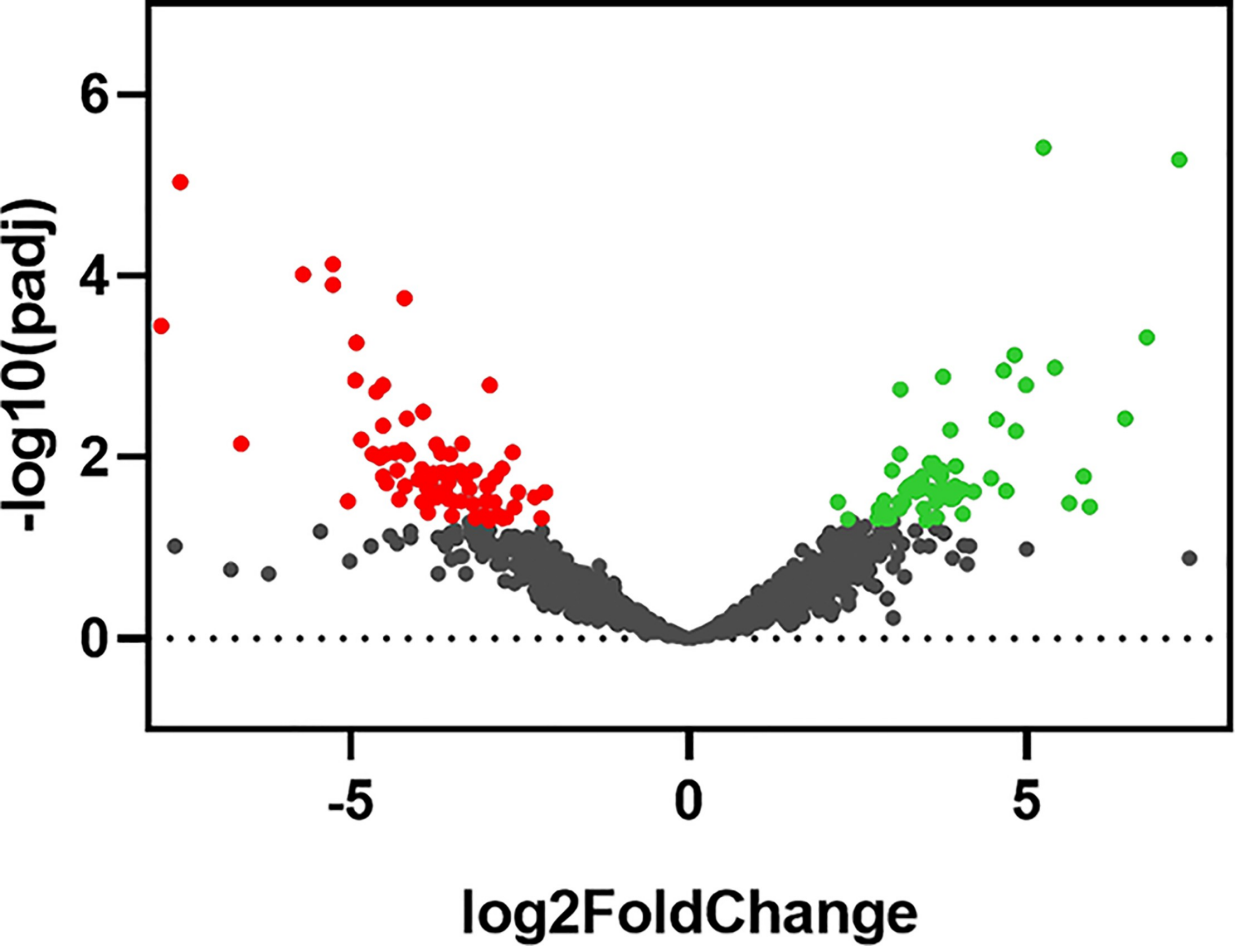
Volcano plot of statistical significance against fold change between diabetic and non-diabetic skin.

**Fig 2:**
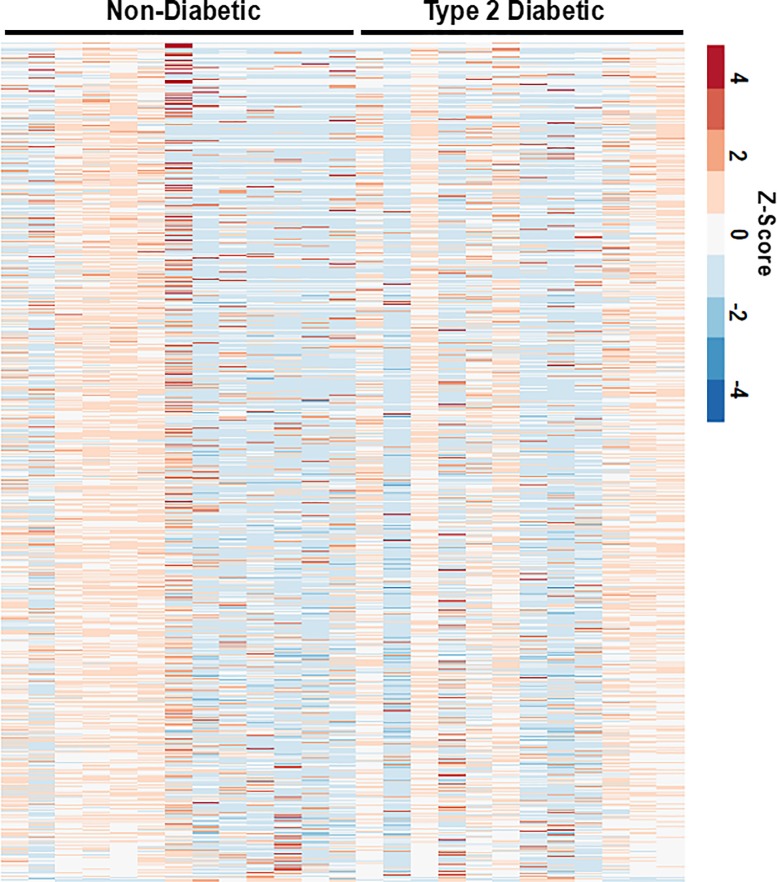
Heat map of the z-score for the top 30% varying genes for diabetic and non-diabetic patients.

**Fig 3:**
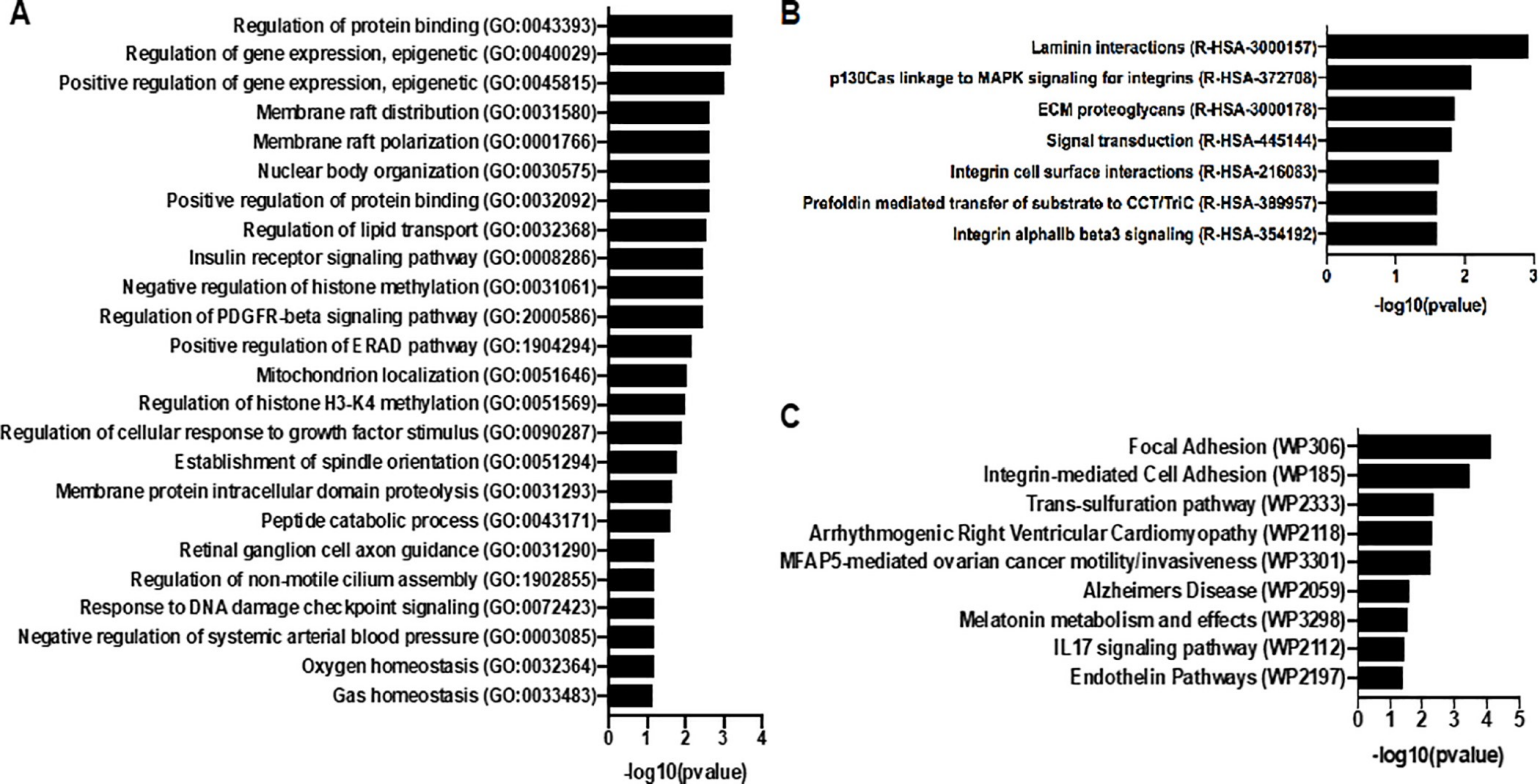
Gene and pathway ontology analysis of significantly regulated genes between diabetic and non-diabetic patient skin samples. (A) Most altered pathways between diabetic and non-diabetic skin using the reactome database analysis. (B) Significantly alter pathways for the gene ontology analysis for biological process when comparing diabetic and non-diabetic patients. (C) Pathways significantly altered using the wikipathways analysis tool comparing diabetic and non-diabetic patients.

Skin tissues were obtained from 13 non-diabetic and 14 type 2 diabetes donors. We examined expression changes of 58,037 transcribed genes. Differential expression testing was performed based on the DESeq2 negative binomial distribution by comparing type 2 diabetic samples to normal samples. We found 64 significantly upregulated genes and 120 downregulated genes (**Fig 1**; **S1 Table**). There was significant patient-to-patient variability in the samples (**Fig 2**). Overall the five most upregulated genes when comparing skin samples from diabetic patients to non-diabetic patients included three non-coding RNA (lncRNA) genes (LINC01118, RP11-545I5.3 and an unknown lncRNA), an enzyme involved in lipid metabolism (ABHD16A) and a potassium channel that is important in smooth muscle tone and nerve function (KCNMA1). Conversely, the most downregulated genes included multiple pseudogenes and lncRNA (LINC01060, HPRT1P2, and CCNYL3) as well as the transcription factor NKX2_1 and a member of the TPD52 protein family associated with proliferation and vesicle trafficking (TPD52L3). Pathway analysis of the significantly altered genes revealed significant alterations in genes relating to epigenetic regulation of gene expression, lipid membrane rafts, growth factor signaling/PDGFRβ signaling, and proteolysis (**Fig 3A**). Gene ontology analysis of the biological processes most regulated in the genes and the functional pathways being regulated supported significant regulation of genes relating to integrin-based adhesion, focal adhesion formation and ECM proteoglycans (**Fig 3B and 3C**).

### Regulation of genes related to transcription and gene regulation

**Fig 4:**
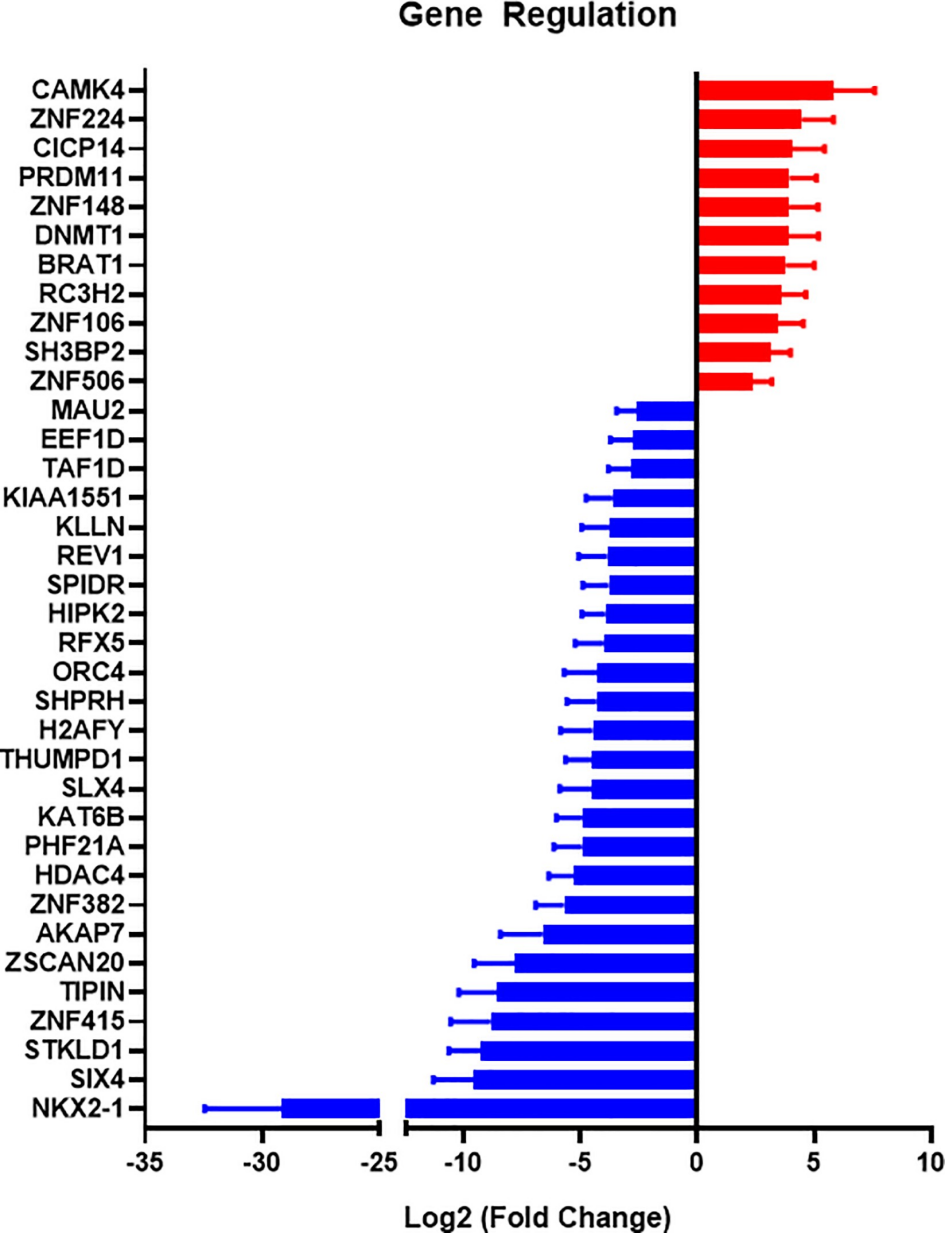
Transcription- and regulation-related genes that were significantly regulated in type 2 diabetic patient skin samples.

The largest category of genes with significant regulation was that involved in gene regulation (**Fig 4**). Among the 11 upregulated genes, five genes (CAMK4, ZNF106, RC3H2, DMNT1, ZNF148) showed some relationship with type 2 diabetes in the previous studies[11–15]. One of the significantly upregulated genes was for calcium/calmodulin-dependent protein kinase (CAMK4). This protein phosphorylates transcription factors and can thereby regulate gene expression. Its target transcription factors include high-mobility group protein 1 (HMGB1), a proinflammatory mediator of chronic pain development[16]. Increased phosphorylation of CAMK4 is seen in a rat dorsal root ganglion after STZ-induced diabetes. [17].

Another notable gene that was upregulated in patients with diabetes was the transcriptional repressor ZNF224. Overexpression of ZNF224 is linked to the reduction of mitochondrial citrate carrier (CIC)[18, 19], a key enzyme in fatty acid and cholesterol synthesis[19]. Rats with STZ-induced diabetes had a reduced CIC activity[20], consistent with our findings.

Among the downregulated genes in our study, seven of these genes were found to also be associated with diabetes in previous studies[21–28]. The most downregulated gene in the gene regulation category was NKX2-1, also known as thyroid transcription factor 1 (TTF1). This transcription factor is involved in differentiation of the thyroid, lung and brain[29] and is linked to neuronal disorders[30, 31]. Mutation of NKX2-1 is also related to the reduction in mitochondrial respiratory chain complex activity[32]. Notably, reduced mitochondrial activity is one of the characteristics of diabetes[33]. Another highly downregulated transcription factor was SIX4, a key transcription factor in the development of sensory neurons and myogenesis[34–36]. Peripheral neuropathy is a common finding in diabetic patients and underlies alterations in biomechanics that leads to increased risk of ulcer formation[37]. In addition, diabetes can cause muscle weakness by altering muscle progenitor cells and other mechanisms[38, 39]. Thus, further investigation of these genes in the context of reduced mitochondrial activity and deficits in neural and muscular healing in diabetes would be merited.

### Regulation of metabolism and mitochondrial related genes

**Fig 5:**
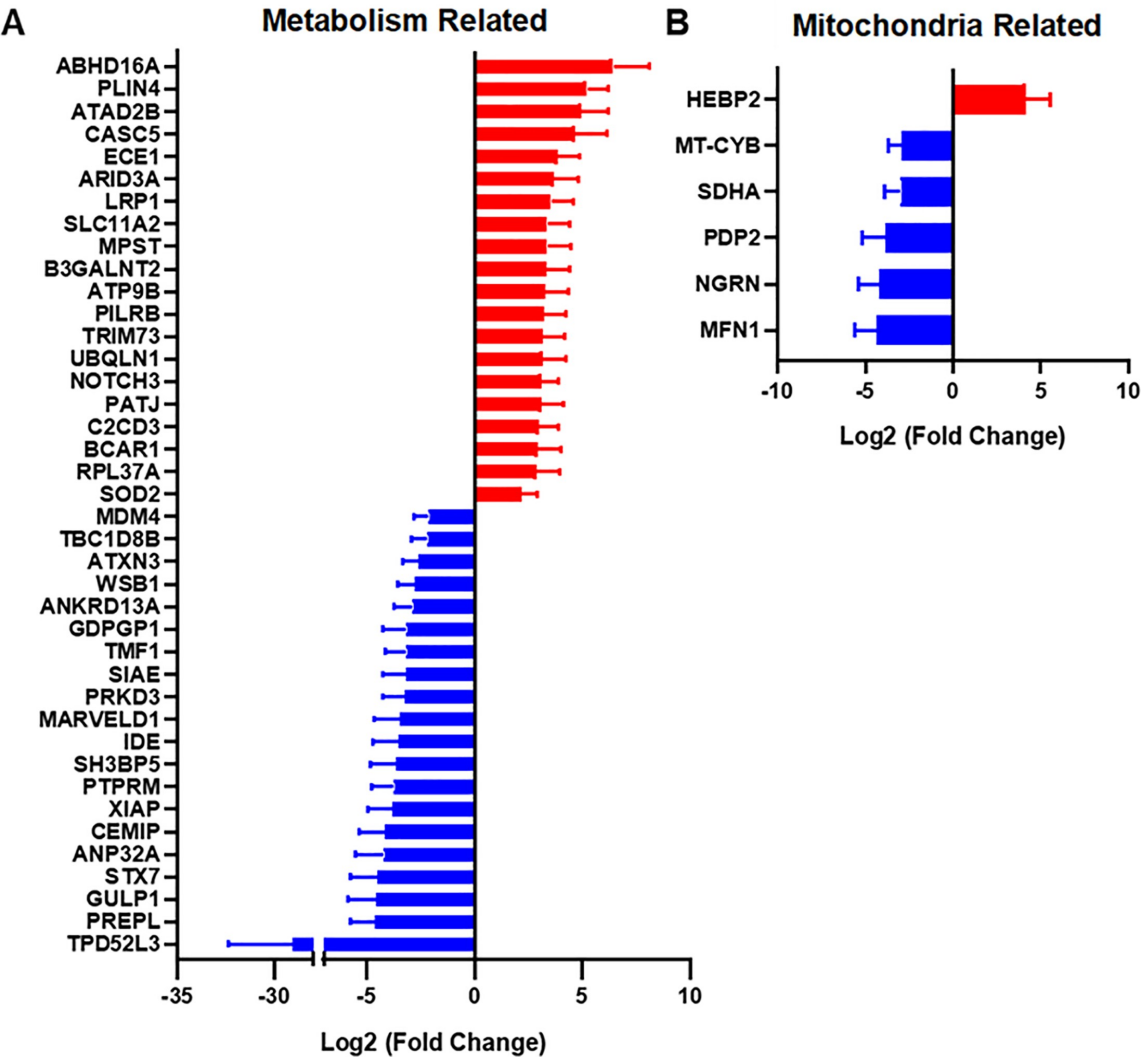
Metabolism genes and mitochondrial genes are significantly upregulated or downregulated in patients with diabetes. (A) Alterations in metabolism-related genes when comparing patients with T2D and non-diabetic patients. (B) Mitochondrial genes that were significantly altered between the patient groups.

We observed significant regulation in a number of genes relating to metabolism (**Fig 5A**). The most upregulated gene in this category was α/β hydrolase domain containing 16A (ABHD16A), which is a member of the α/β hydrolase domain-containing (ABHD) protein family and is expressed in dendritic cells, macrophages, lymphocytes, and lymphoid organs. Interplay between ABHD16A and ABHD12 dynamically regulates immunomodulatory lysophosphatidylserines and can alter the release of proinflammatory cytokines from macrophages[40]. Other studies found several associations between ABHD16A and the genetic predisposition to coronary artery aneurysm and Kawasaki disease[41].

We also found an 11.8 fold increase in gene expression for LDL Receptor Related Protein 1 (LRP1) in skin from diabetic patients in comparison to control patients. LRP1 interacts with many ligands including lipoproteins, extracellular matrix proteins, protease, cytokines and growth factors. Biological roles of LRP1 covers broad ranges of activities such as lipid metabolism, cell growth, migration and tissue invasion, but most notable role of LRP1 in terms of wound healing should be extra cellular matrix (ECM) remodeling. A recent study found that LRP1 markedly inhibited fibronectin remodeling by regulating cell-surface urokinase receptor and plasminogen activation[42]. This process may be relevant to reduced healing of wounds and altered ECM remodeling observed in diabetic patients. Another study found upregulation of LRP1 in the epicardial fat tissue from patients with type 2 diabetes[43]. Interestingly, GULP1, a ligand that binds to LRP1, was significantly decreased in patients with type 2 diabetes in our study. GULP1 is an adapter protein necessary for the engulfment of apoptotic cells by phagocytes[44] and also functions as a modulator of glycosphingolipid and cholesterol transport[45].

We also found an 8.7 fold increase in NOTCH3 expression in skin from patients with type 2 diabetes. The Notch3 protein plays a key role in the function and survival of vascular smooth muscle cells, and is essential for the maintenance of blood vessels[46]. Recently, several studies found that NOTCH3 polymorphism seems to be a risk factor for both ischemic disease and diabetes. For example, NOTCH3 381C>T and 1735T>C polymorphisms found in the peripheral blood were associated with ischemic stroke and to be the risk factors for ischemic stroke[47]. The C381T (rs3815188) variants in exon 3 and A684G (rs1043994) variants in exon 4 of the NOTCH3 gene in the peripheral blood were also strongly associated with type 2 diabetes[48].

Superoxide dismutase 2 (SOD2) was increased 4.6-fold in patients with type 2 diabetes in our study. This gene encodes a mitochondrial protein, also known as manganese superoxide dismutase (MnSOD), that is a manganese-dependent enzyme that acts on superoxide produced as a byproduct of oxidative phosphorylation. SOD2 converts superoxide into hydrogen peroxide, which can be further detoxified by other enzymes. This enzyme is induced by oxidative stress and is increased in chronic ischemic wound models[49, 50]. In addition to its role in protecting oxidative damage, SOD2 also regulates signaling through reactive oxygen species (ROS)[51]. Low levels of SOD2 impair wound healing and enhancing SOD2 expression/activity enhanced wound repair[52, 53]. Polymorphisms in the SOD2 gene are associated with type 2 diabetes in the Japanese American population. The A16V polymorphism of SOD2 was seen frequently in the peripheral blood of type 2 diabetes patients, and study confirmed that this polymorphism decreases SOD2 activity, which successively will increase oxidative stress[54]. The C47T polymorphism in SOD2 gene has also been associated with a protective effect against diabetic microvascular complications[55].

The most downregulated gene in the metabolism category was tumor protein D52-like family of proteins (TPD52L3; **Fig 3A**). This gene has not been linked to type 2 diabetes or wound healing yet, but a study found that exogenous expression of human TPD52 in cultured cells resulted in significantly increased numbers of lipid droplet.[56] Lipid droplets store excess fatty acids within adipocytes. Thus, TPD52L3 seems to be associated with excess lipid storage in adipose cell. We hypothesize that lowering of TPD52L3 in type 2 diabetes, may lead to alterations in lipid storage and altered function of adipocytes.

Previous studies have linked diabetes to the development of mitochondrial dysfunction[57]. For example, point mutations resulting in amino acid substitutions in MT-CYB (D214N) showed defective mitochondrial ATP production[58–60]. Therefore, it is imperative to examine how diabetes affects the expression of mitochondrial related genes. The only significantly upregulated mitochondrial gene was HEBP2, which codes for the Heme Binding Protein 2 (**Fig 5B**). The protein encoded by this gene plays a role in the loss of mitochondrial membrane potential prior to cell death in necrosis. Methylation on this gene has been found in diabetic patients[61, 62]. The most strongly downregulated mitochondrial gene was MFN1, which codes for the mitofusin-1 protein. This protein is expressed on the mitochondrial membrane and is involved in the regulation of mitochondrial fusion. A study showed that type 2 diabetes was related to mitochondrial network fragmentation in myocardium and a large decrease in MFN1 expression[63]. Interestingly, mice with MFN1 knockout displayed a higher preference for lipid use as energy substrate[64]. Among five downregulated genes, three genes were downregulated in previous studies, including PDP2, NGRN, and MFN1[63, 65].

### Alterations in pseudogene, lncRNA, RNA gene, RNA processing genes

**Fig 6:**
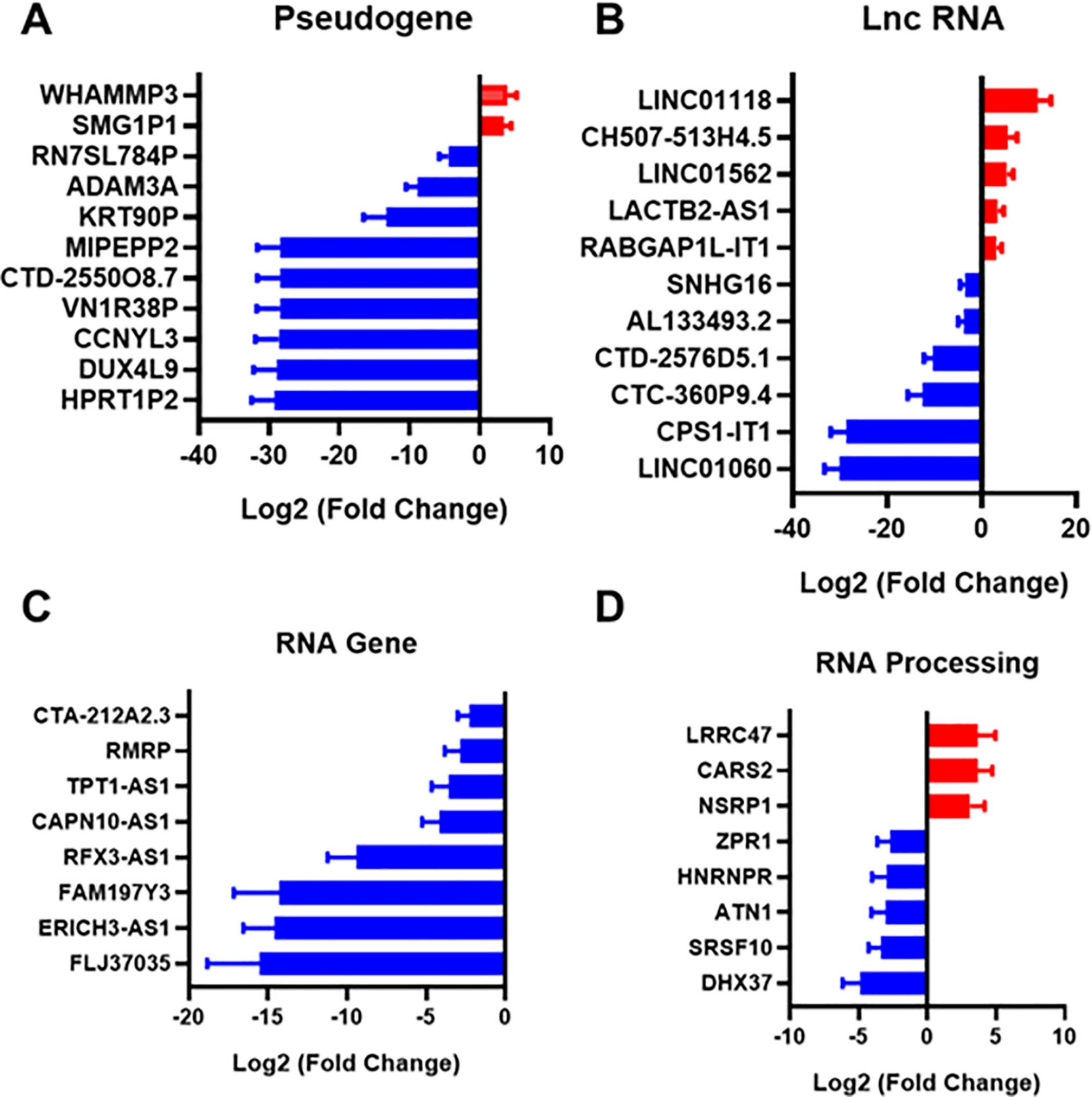
Pseudogene, lncRNA, RNA gene, and RNA processing genes are significantly upregulated or downregulated in patients with diabetes. (A) Pseudogenes that were altered inin skin from patients with type 2 diabetes. (B) Long non-coding RNAs (lncRNAs) that were altered in the skin of patients with type 2 diabetes (C) RNA genes with significant regulation in comparing the two patient groups. (D) Genes involved in RNA processing that were significantly changed between diabetic and non-diabetic patient groups.

A pseudogene is a DNA sequence that is an imperfect copy of a functional gene. Pseudogenes were once thought to be junk DNA, but it has since been recognized that some pseudogenes play essential roles in gene regulation of their parent genes[66]. We found two genes upregulated and nine genes downregulated in the pseudogene category (**Fig 6A**). Putative Cyclin-Y-Like Protein 3 is a pseudogene for cyclin-dependent protein serine/threonine kinase. The function of CCNYL3 is not yet fully understood, but overall it is expected to relate to the regulation of cell proliferation. Double Homeobox 4 Like 9 (DUX4L9), also known as DUX4c, is a pseudogene that has similarity to DUX4 transcription factor that was suggested to be a risk gene to cause facioscapulohumeral muscular dystrophy[67]. This gene rapidly downregulates the transcription factor Myogenic Differentiation 1 (MyoD), resulting in impaired myogenic differentiation and muscle regeneration[68]. Low expression of DUX4 impairs myogenesis through a reduction in myogenic gene expression[69]. DUX4L9 expression impairs myofibrillogenesis and potentially has a role in controlling muscle cell differentiation through association with type III intermediate filament protein desmin[70, 71].

Recent studies showed that deregulation of lncRNAs is associated with various diseases such as cancer, Alzheimer’s disease, and heart disease[72–76]. We found five upregulated genes and six downregulated genes under the long non-coding RNA (lncRNA) category (**Fig 6B**). Within the lncRNA category, CPS1-IT1 seems to be involved in type 2 diabetes. In the previous study, an impairment in neovascularization results from a high glucose induced defect in transactivation of hypoxia-inducible factor-1α (HIF-1α), and HIF-1α is controlled by CPS-IT1[77, 78]. The lncRNA LINC01118 was the most upregulated lncRNA in patients with type 2 diabetes in our study. LINC01118 has been associated with chemoresistance and increased cell migration in cancer[79]. The lncRNA LINC01060 was strongly decreased in our study in patients with type 2 diabetes. This lncRNA has been associated with poor prognosis in pancreatic cancer and inhibited pancreatic cancer proliferation and invasion[80]. Thus could also potentially contribute to poor wound healing prognosis.

In the RNA gene category, we found eight downregulated genes (**Fig 6C**). One of the downregulated RNA genes was Calcium-Activated Neutral Proteinase 10 Antisense 1 (CAPN10-AS1). In a previous study, CAPN10 expression was elevated in a pancreatic islet from type 2 diabetes[81]. It is generally known that antisense interferes the transcription of paired RNA (sense RNA)[82]. We also found the RNA gene ERICH3-AS1 to be highly downregulated in patients with type 2 diabetes. Consistent with our findings, this gene is downregulated in a sciatic nerve in diabetic mice[83]. Little is known about the function of ERICH3, however it is highly expressed in airway cilia and has been associated with major depressive disorder[84, 85].

In the RNA processing category **(Fig 6D)**, we found a downregulation of Zinc Finger Protein 1 (ZPR1). The ZPR1 binds to the promoter of peroxisome proliferator-activated receptor gamma (PPARG) proteins 1 and 2, which play a key role in insulin sensitivity and obesity[86, 87]. A previous study reported the evidence linking the genetic susceptibility of common variants in *ZPR1* to type 2 diabetes with the levels of fasting plasma glucose and blood hemoglobin A1c, suggesting *ZPR1* might be involved in abnormal glucose metabolism[88]. However, the expression tendency seems to be different in skin tissue and others. ZPR1 mRNA in the brain is upregulated in mice fed a high-fat diet[89], whereas our result showed downregulation in the skin.

Several other notable genes were regulated in the RNA processing category including cysteinyl-tRNA synthetase 2 (CARS2), Heterogeneous Nuclear Ribonucleoprotein R (HNRNPR), and DEAH-box helicase 37 (DHX37). CARS2 plays a critical role in protein biosynthesis by charging tRNAs with their cognate amino acids. In a previous study, CARS2 was upregulated in a muscle tissue of patients with type 2 diabetes[90]. Heterogeneous Nuclear Ribonucleoproteins are RNA binding proteins and associated with pre-mRNAs in the nucleus. HNRNPR appears to influence pre-mRNA processing and other aspects of mRNA metabolism and transport. HNRNPR was downregulated in the islet-specific CD4 + T cells in type 1 diabetes -susceptible NOD mice[91]. DHX37 encodes a DEAD box protein. DEAD box proteins are characterized by the conserved motif Asp-Glu-Ala-Asp (DEAD), and implicated in alteration of RNA secondary structure such as translation initiation, nuclear and mitochondrial splicing, and ribosome and spliceosome assembly. In the previous study, DHX37 was downregulated in rat corneal epithelia in type 1 diabetes[27, 92]. Our result also showed a significant downregulation of DHX37, implying the malfunction in constructing RNA secondary structures may occur in the diabetic skin or corneal epithelia.

### Cytoskeleton, membrane, and adhesion related genes

**Fig 7:**
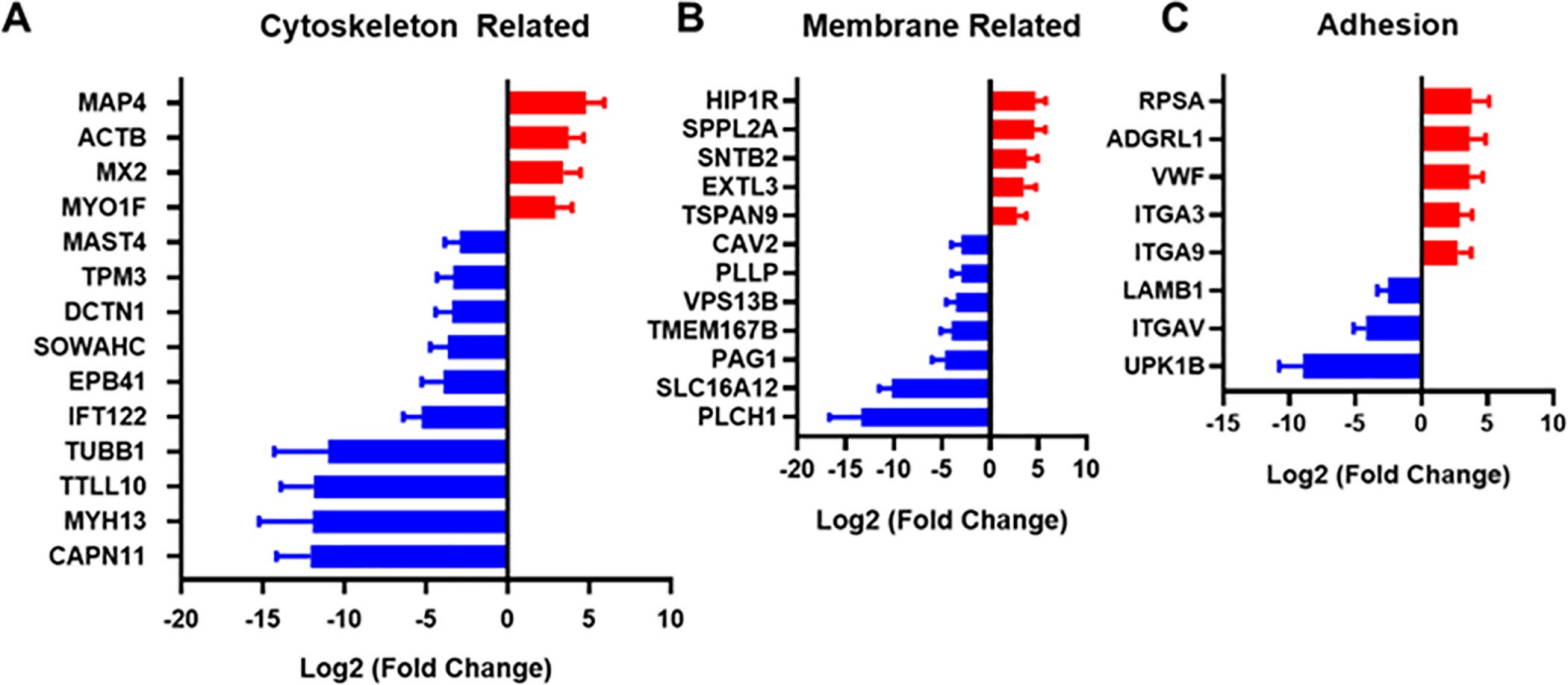
Cytoskeleton, membrane and adhesion genes are significantly upregulated or downregulated in patients with diabetes. (A) Cytoskeletal genes altered in the skin in patients with type 2 diabetes. (B) Membrane related genes that were significantly different between diabetic and non-diabetic patients. (C) Cell adhesion related genes that were altered in type 2 diabetes.

Elevated glucose levels can alter the mRNA and protein expression of contractile smooth muscle markers[93]. **Fig 7A** shows significantly up and downregulated cytoskeleton related genes in type 2 diabetic skin. We found β-actin (ACTB) to be significantly upregulated in the skin of type 2 diabetic patients. We also found increases in the Myosin-IF (MYO1F) gene. MYO1F exacerbates atherogenesis and is reported to be a biomarker candidate for patients with obstructive coronary artery disease or advanced atherosclerosis[94]. Among downregulated genes, Tropomyosin (TPM3) was downregulated ten-fold in the type 2 diabetic test group. This gene is translated into α-tropomyosin, which controls contraction in type I skeletal muscle fibers. Consistent with our findings, a previous study showed the downregulation of TPM3 in rats with alloxan-induced diabetes[95].

We found 12 genes in this category that were significantly altered in the skin of diabetic patients in comparison to control patients **(Fig 7B)**. Cell membrane lipid composition can alter the effectiveness of glucose transporters and has been implicated in the microvascular pathophysiology in diabetes[96]. The most upregulated gene in our study was the huntingtin interacting protein 1 related protein (HIP1R). This protein serves an important role in clathrin mediated endocytosis[97]. A recent study revealed its function in β-cell survival and glucose-stimulated insulin secretion, and HIP1R is downregulated in type 1 diabetes[98]. On the other hand, HIP1R expression is increased in the pancreas in type 2 diabetes[99].

We found a significant decrease in plasmolipin (PLLP) in the skin of patients with type 2 diabetes. A recent study showed that Notch signaling is mediated by PLLP and STX7 in epithelial cells: silencing STX7 impairs activation of Notch through PLLP[100, 101]. In our study, both of STX7 and PLLP were significantly downregulated in diabetic patient skin. The Notch1 pathway is a key regulator of wound healing[102]. Thus, defects in this pathway may be potential targets for improving healing in diabetes.

Tissue repair or wound healing process requires the regulation of cell adhesion molecules to control proliferation or migration of cells. The most upregulated gene in this category was RPSA, which codes for the Laminin Receptor 1 (**Fig 7C**). This gene is differentially expressed in wound margins at different sites on the body and has a role in adhesion and migration in the intestinal epithelia[103, 104]. We also observed increases in the genes for integrin α3 and α9, as well as a decrease in gene expression for integrin αv. Integrin α3β1 is suppressed by α9β1 1 during the resolution wound angiogenesis[105]. In one study, integrin α3β1 also inhibited re-epithelialization in wound healing[106]. Another study demonstrated that ITGA3 knockout mice had inhibited wound healing and that integrin α3β1 served as an inhibitor of Smad7 during the wound healing process[107]. Integrin αv is overexpressed in endothelial cells during the formation of new blood vessels and is a key adhesion receptor in many steps of angiogenesis[108]. In addition, αv integrins have a role in wound healing through the regulation of TGF-β and regulation of cell migration[109]. We also found an increase in von Willebrand factor (vWF) gene expression in patients with type 2 diabetes. The protein is involved in platelet adhesion and has a role in vascular inflammation and thrombosis[110]. There have been previous studies showing that high levels of VWF are associated with type 2 diabetes[111–113]. Our study adds to these findings in showing a significant upregulation in skin from diabetic patients.

### Calcium signaling and inflammation related genes

**Fig 8:**
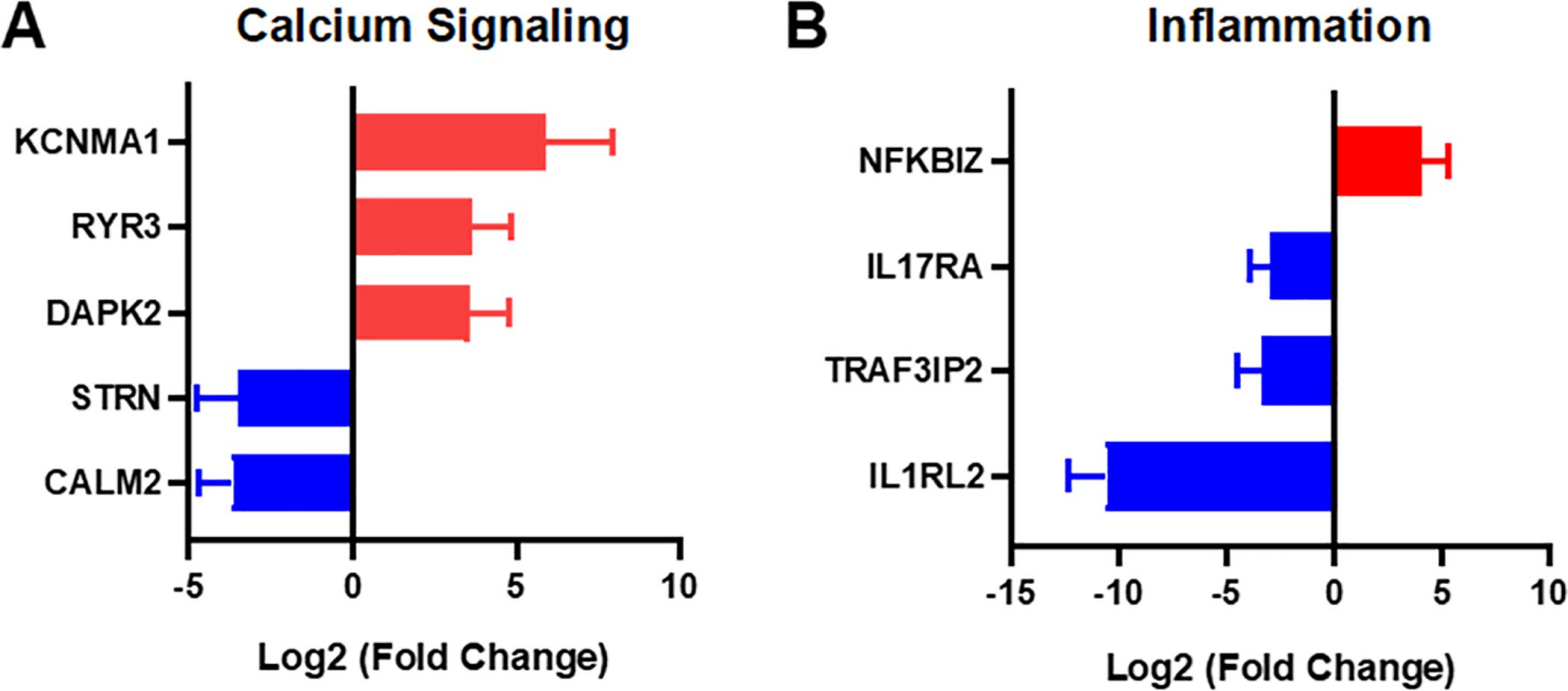
Inflammation and calcium signaling related genes that are significantly upregulated or downregulated in patients with diabetes. (A) Calcium signaling related genes that were significantly altered between the patient groups. (B) Inflammation-related genes that were significantly different between the non-diabetic and type 2 diabetic groups.

In the calcium-signaling gene category, we found a large increase in expression of KCNMA1 in patients with type 2 diabetes (**Fig 8A**). This gene encodes the calcium- and voltage-dependent potassium channel KCa1.1. This channel has been implicated in rheumatoid arthritis, the regulation of integrins, and myoblast function[114–116]. We also found decreases in expression for the genes for striatin (STRN) and calmodulin 2 (CALM2). Calmodulin 2 is involved in regulating a large number of proteins through calcium-mediated mechanisms and striatin is a calmodulin binding protein.

Among the significantly regulated genes to inflammation, we found a significant increase in the gene expression NF-κB inhibitor zeta (NFKBIZ) in patients with type 2 diabetes (**Fig 8B**). The protein encoded by this gene inhibits NF-κB, a key transcription factor in inflammation. We also found three significantly downregulated inflammatory genes including a receptor for IL-17 family members (IL17RA), TRAF3 Interacting Protein 2 (TRAF3IP2) and Interleukin 1 Receptor Like 2 (IL1RL2; IL-36 receptor). Interleukin-17A is a proinflammatory cytokine that promotes the recruitment of neutrophils during wound healing. In wound healing, it likely delays wound healing while enhancing an inflammatory response that would promote the removal of microbes[117]. TRAF3IP2 alters keratinocyte differentiation in the skin and inhibits response to IL-17[118]. Both IL-17A and tumor necrosis factor–α (TNF-α) are induced by IL-36 cytokines in keratinocytes[119]. The cytokine IL-36γ promotes wound closure through a mechanism involving toll-like receptor 3 (TLR3), TIR-domain-containing adapter-inducing IFN-β (TRIF) and the transcription factor SLUG[120]. Thus, our findings suggest that IL17/IL36 mediated inflammatory pathways may be altered in diabetic skin. Indeed, the IL17/IL36 signaling axis is associated with psoriasis and targeted therapies are showing success. Psoriatic skin has hallmarks to some aspects of the hyperproliferative status of chronic non-healing wounds and comparisons between these established pathways are worthy of further investigation. We have summarized a comparison of our results to others in previous studies in **Table 2**[121–123].

**Table 2:** Comparison of study results with previous studies.

Gene	This Study	Previous Studies	Model animal	Reference
CAMK4	Increase in T2D	Increased phospho-CAMK4	STZ-Induced Diabetes in Rat	17
ZNF224	Increase in T2D	Reduced CIC activity	STZ-Induced Diabetes in Rat	20
ABHD16A	Increase in T2D	Genetic predisposition to coronary artery aneurysm and Kawasaki disease	Human	41
LRP1	Increase in T2D	Upregulation in the epicardial fat tissue	T2D in Human	43
NOTCH3	Increase in T2D	NOTCH3 polymorphism is a risk factor for ischemic disease and diabetes	Ischemic Stroke in Human	47, 48
SOD2	Increase in T2D	SOD2 polymorphism is assocated with T2D	T2D in Human	54
HEBP2	Increase in T2D	Methylation on this gene is found in T2D	T2D in Human	61, 62
CARS2	Increase in T2D	Upregulated in muscle tissue	T2D in Human	90
MYO1F	Increase in T2D	MYO1F exaerbates atherogenesis	Coronary Artery Disease in Human	94
HIP1R	Increase in T2D	Downregulated in β-cell in T1D, but upregulated in the pancreas in T2D	T1D/T2D in Human	98, 99
vWF	Decrease in T2D	High levels of vWF	T2D in Human	111–113
NKX2-1	Decrease in T2D	Mutation in NKX2-1 links to reduced mitochondrial activity	Human	32
MFN1	Decrease in T2D	Downregulated	T2D in Human	63
PDP2	Decrease in T2D	Decreased protein expression	T2D in Human	65
NGRN	Decrease in T2D	Downregulated	T2D in Human	63
CAPN10	Decrease in T2D	Upregulated in pancreatic islet	T2D in Human	81
ERICH3-AS1	Decrease in T2D	Downregulated in a sciatic nerve	Diabetic Mice	83
ZPR1	Decrease in T2D	Upregulated in brain	High-Fat Diet Fed Mice	89
HNRNPR	Decrease in T2D	Downregulated in the islet-specific CD4 + T cells	T1D-Susceptible NOD Mice	91
DHX37	Decrease in T2D	Downregulated in corneal epithelia	T1D Rat	27, 92
TPM3	Decrease in T2D	Downregulated	Alloxan-Induced Diabetes in Rat	95

## Conclusions

We examined the gene expression in the skin from patients with type 2 diabetes and non-diabetic patients. Among the genes regulated there were many that had been identified as being modulated in diabetic animals and this study serves to provide confirmation that these changes also occur in the skin of human patients. This information could be useful in identifying the causal factors and potential therapeutic targets for the prevention and treatment of diabetic related diseases.

## Supporting information

S1 Fig Volcano plot of statistical significance against fold change between diabetic and non-diabetic skin, demonstrating 64 significantly upregulated genes and 120 downregulated genes. (TIF)

S1 Table (XLSX)
