# Supplementary figures and images for: Genome wide analysis of gene expression changes in skin from patients with type 2 diabetes
Source: PLoS One. 2020 Feb 21;15(2):e0225267. doi: 10.1371/journal.pone.0225267 (PMC7034863; doi:10.1371/journal.pone.0225267)

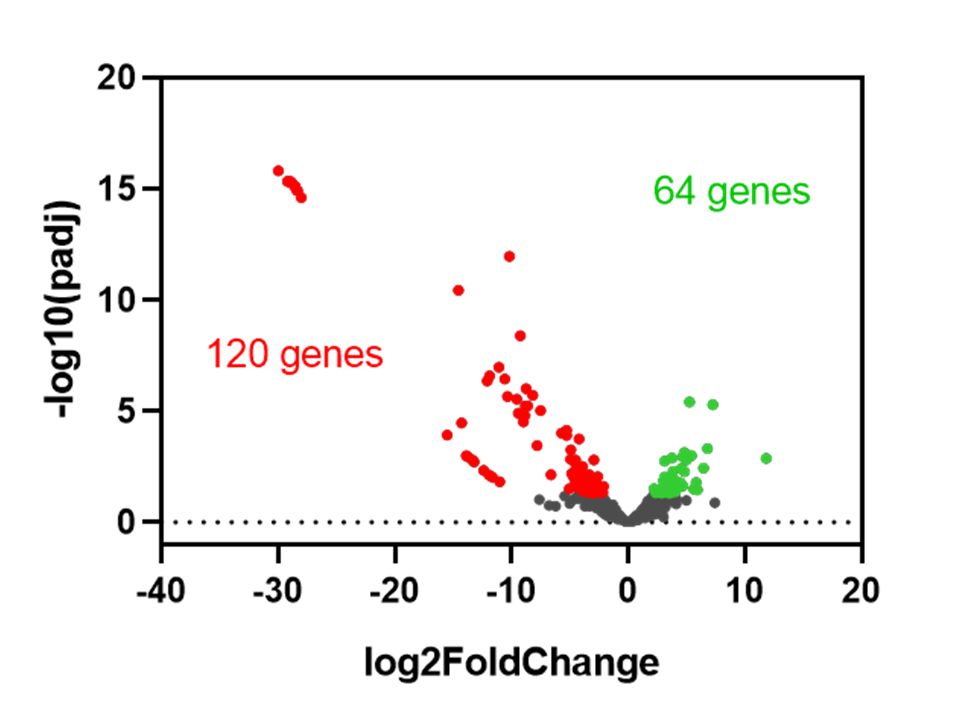

Supplement: S1 Fig — (TIF) [file pone.0225267.s001.tif]
